# Bone Disease in Newly Diagnosed Lupus Nephritis Patients

**DOI:** 10.1371/journal.pone.0106728

**Published:** 2014-09-17

**Authors:** Aline Lázara Resende, Luciene Machado dos Reis, Cristiane Bitencourt Dias, Melani Ribeiro Custódio, Vanda Jorgetti, Viktoria Woronik

**Affiliations:** Nephrology Division, Sao Paulo University Medical School, Sao Paulo, Brazil; Universidade de Sao Paulo, Brazil

## Abstract

**Introduction:**

Bone loss in Lupus Nephritis (LN) patients is common and multifactorial. The aim of this study was to evaluate the bone status of newly diagnosed LN patients and their correlation with inflammatory factors involved in LN physiopathology.

**Methods:**

We studied 15 pre-menopausal patients with ≤2 months of diagnosed SLE and LN. Patients with prior kidney or bone disease were excluded. In addition to biochemical evaluation (including 25-hydroxyvitamin D_3_ [25(OH)D] and Monocyte Chemotactic Protein (MCP1) dosage), we performed bone biopsies followed by osteoblast culture, histomorphometric and immunohistochemistry analysis.

**Results:**

LN patients presented a mean age of 29.5±10 years, a proteinuria of 4.7±2.9 g/day and an estimated glomerular filtration rate (GFR) of 37(31–87) ml/min/1,73 m^2^. They were on glucocorticoid therapy for 34±12 days. All patients presented vitamin D insufficiency (9.9±4.4 ng/ml, range 4–20). Urinary MCP1 correlated negatively with 25(OH)D (r = −0.53, p = 0.003) and positively with serum deoxypyridinoline (r = 0.53, p = 0.004). Osteoblasts isolated from LN bone biopsies presented a significantly higher expression of MCP-1 when compared to controls (32.0.±9.1 vs. 22.9±5.3 mean fluorescence intensities, p = 0.01). LN patients presented a significantly reduced osteoid volume, osteoid thickness, osteoid surface, mineralization surface and bone formation rate, associated with an increased eroded surface and osteoclast surface. Patient’s bone specimens demonstrated a reduced immunostaining for osteoprotegerin (0.61±0.82 vs. 1.08±0.50%, p = 0.003), and an increased expression of Receptor Activator of NF-κB ligand (RANKL) (1.76±0.92 vs. 0.41±0.28%, p<0.001) when compared to controls.

**Discussion:**

Newly diagnosed LN patients presented a significant disturbance in bone metabolism, characterized by an impaired bone formation and mineralization, associated with an increase in resorption parameters. Glucocorticoid use, vitamin D insufficiency and inflammation might be involved in the physiopathology of bone metabolism disturbance.

## Introduction

Systemic Lupus Erythematosus (SLE) is a chronic autoimmune disease that may compromise multiple organs. Although several mechanisms may influence the loss of self-tolerance, cytokines are thought to initiate and amplify inflammation and organ damage [1]. Because disease survival has improved over the last decades, scientific community has placed a growing emphasis on prevention and treatment of SLE-related comorbidities. Bone loss is not only a common (with osteopenia and osteoporosis been reported in 25–74% and 1.4–68% of patients, respectively), but also a potentially preventable condition associated with SLE [2], [3].

The etiology of bone loss in SLE is probably multifactorial, involving both traditional and disease-related risk factors [3]. Glucocorticoid use has been extensively associated with reduced bone mineral density (BMD) in SLE patients [4]–[7]. Indeed, Glucocorticoid-induced bone disease (GIO) is considered the most common cause of secondary osteoporosis. The loss of BMD in GIO is biphasic: it occurs rapidly (6–12% loss) within the first year and more slowly (3% loss yearly) thereafter [8]. However, the risk of fractures seems to increase 75% within the first 3 months of therapy, before a substantial decline in BMD [9], and possibly due to the loss of bone strength [10]. It is not clear the minimum dose and duration of glucocorticoid therapy that could increase the risk of fracture. Guidelines from the American College of Rheumatology, the National Osteoporosis Foundation and the Royal College of Physicians agree that pharmacologic therapy should be used in patients exposed to glucocorticoid for at least 3 months [11]–[14]. Nevertheless, the contribution of corticosteroid therapy to bone loss in SLE remains unclear as several studies found no association between reduced BMD and corticosteroid therapy [15]–[20]. Furthermore, some investigators have reported a relationship between SLE and lower BMD in patients never receiving corticosteroids [19], [21], [22], suggesting that SLE activity *per se* may be a risk factor for bone loss.

Based on animal and *in vitro* observations [23]–[27], recent studies have raised the attention on the interplay between inflammatory factors and bone remodeling and metabolism not only in SLE [2], [16], [28], [29], but also in other clinical conditions, such as Rheumatoid Arthritis, Inflammatory Bowel Disease, Idiopathic Hypercalciuria and Estrogen withdrawal [24], [26], [30]–[34]. There are many evidences that the immune and skeletal systems not only share a number a regulatory molecules (such as cytokines, receptors, signaling and transcription factors), but also interact in the bone marrow [35].

Lupus nephritis (LN), a major clinical manifestation of SLE, is notably characterized by an intense inflammatory activity. An important mediator of renal injury is a leukocyte chemotactic factor called Monocyte chemoattractant protein-1 (MCP-1). Noteworthy, the urinary levels of MCP-1 are widely recognized as a sensitive and specific biomarker of LN activity [36]–[41]. Besides inflammation, LN patients may confront an increased risk for bone disturbances due to proteinuria and/or kidney dysfunction, usually aggravating the vitamin D insufficiency [2], [16], [42], [43].

Finally, literature data on SLE-related bone disease are focused in bone mineral density loss (BMD) measured by the most widely used technique known as DEXA (Dual Energy Xray Absorptiometry) [7], [19], [20], [22], [44]–[46]. Although clinically relevant, the decreased BMD might not reflect the bone strength and especially does not help to understand the physiopathology of the bone disorder [10], [47]. The aim of this study was evaluate the bone status of newly diagnosed lupus nephritis patients and their correlation with inflammatory factors thought to be involved in LN physiopathology. In addition to biochemical evaluation, we performed bone biopsies followed by osteoblast culture, histomorphometric and immunohistochemistry analysis.

## Methods

### Subjects

We studied pre-menopausal patients with ≤2 months of diagnosed SLE and LN (according to the American College of Rheumatology classification criteria [48] attended in Sao Paulo University Medical School from December 2010 to December 2012. Patients with prior kidney disease, pregnancy, thyroid dysfunction, currently use of calcium supplements, anticonvulsants, oral anticoagulants, or other drugs affecting bone metabolism were excluded. The local ethics committee of the Sao Paulo University Medical School approved the study protocol, and patients provided written informed consent.

Clinical activity was estimated according to the Systemic Lupus Erythematosus Disease Activity Index (SLEDAI) [49]. Kidney biopsies were performed and LN was classified according to the current International Society of Nephrology (ISN)/Renal Pathology Society classification [50] by an independent pathologist. All patients received glucocorticoids since they were diagnosed. Other immunosuppressive medications were decided by the clinical staff. Of note, proliferative LN patients were treated with monthly intravenous cyclophosphamide (n = 11) or with oral mycophenolate (n = 2), while Membranous LN (n = 1) was treated with oral cyclosporine. Class II LN (n = 1) was treated with a short course of corticosteroid only.

### Biochemical analysis

Fasting serum and plasma, 24-hour urine, and early-morning spot urine were all collected at hospital admission (on average three weeks before the bone biopsy). Parathyroid hormone (PTH) was measured by immunochemiluminensce [reference values (RV): 16–87 pg/ml] and 25-hydroxyvitamin D_3_ [25(OH)D] by chemoimmunoassay (RV: 30–100 ng/ml). Total calcium (RV: 8.6–10.2 mg/dL), phosphorus (RV: 2.7–4.5 mg/dL) and alkaline phosphatase (RV: 35–104 U/L) were determined by automated routine methods. The Glomerular Filtration Rate (GFR) was estimated by the MDRD simplified formula [51].

The levels of Monocyte Chemotactic Protein (MCP-1) were measured in serum and urine by ELISA assay according to the manufacturer’s directions (Human MCP1 ELISA, R&D Systems). Urinary levels of MCP-1 were standardized to urine creatinine (Cr) measured in the same spot urine and expressed as pg/mg Cr.

We also measured the serum levels of Interleukin (IL) 6 and Tumor Necrosis Factor (TNF) α (Human IL-6 and Human TNFα, both from R&D Systems, Minneapolis, MN, USA), considered biomarkers of SLE activity [52]–[54]. Bone resorption marker deoxypyridinoline was determined by the enzyme-linked immunoassay (Total Deoxypyridinoline ELISA, Quidel, San Diego, CA, USA).

Controls for biochemical analysis were age-matched healthy women, mostly students and employees from our laboratory. Control’s samples were taken at the end of winter (September of 2012), period of the year when lowest vitamin D levels are expected. All patients and controls were permanent residents in metropolitan area of Sao Paulo, a city with subtropical climate located at 23°34′S [55], [56].

### Bone biopsy

After pre-labeling with tetracycline (20 mg/kg/day during 3 days, administered in two time-spaced doses, separated by an interval of 10 days), LN patients were submitted to local anesthesia and light sedation. Bone samples of approximately 1.5 cm in length were obtained from the anterior iliac bone using an electric trephine (Gaulthier Medical, Rochester, MN, USA). Bone specimens were placed in Dulbecco’s Modified Eagle’s Medium (DMEM) supplemented with HEPES 25 mM, penicillin 250 UI, streptomycin 250 µg/ml and 3 µg/ml of amphotericin B, and divided in 2 parts, one for in vitro analysis, as described next, and the another one for bone histology evaluation (histomorphometry) and immunohistochemistry.

### Osteoblast culture

For *in vitro* analysis cancellous bone obtained from bone biopsy were minced into small fragments (1-mm3) that were washed with phosphate buffered saline to remove hematopoietic marrow cells. The bone fragments were first cultured in 100 mm Petri polystyrene dishes (Nunc, Ilinois, USA) with 10 ml of Dulbecco’s Modified Eagle’s Medium, supplemented with antibiotics (penicillin 50 UI, streptomycin 50 µg/ml, amphotericin B 2.5 µg/ml) and 20% fetal bovine serum (Cultilab, Campinas, Brazil). Culture plates were incubated at 37°C in a humidified atmosphere of 95% of air, 5% CO_2_.

The fragments stayed undisturbed for at least 3 days. The medium was replaced three times per week. Outgrowths of spindle-shaped cells became visible in one week or more. After approximately fourteen days, the small fragments were removed and transferred to another Petri dish to continuing primary explant culture. This procedure was repeated three more times to obtain more cells in the first and second passage. Bone cells were cultured until they reached 90% confluence. Bone cells obtained from outgrowths were harvested with trypsin TrypLE Express (Gibco, USA) and separated into three aliquots for cell proliferation, citometry flow experiments, and cryopreservation storage. As the phenotypic expression of human bone cells has been shown to decrease after multiple passages [57], we studied these cells in the first or second passage. The cell population obtained from the trabecular bone surfaces exhibited an osteoblast phenotype, as evidenced by the expression of an early stage marker alkaline phosphatase and the induction of mineralization [58] (data not shown).

Control bone cells were obtained in parallel with the study period from cadaveric age-matched female donor organs (n = 9).

### 1. Cell proliferation

Bone cells were cultured in 24-well plates (1.8×10^4^cells/well) with complete culture medium until they reached 80–90% of confluence. Then, we used serum free medium with ascorbic acid (100 µg/ml) for 16 hours. After this period, we added 5 µCi/ml of Thymidine [Methyl -^3^H] (Amersham Pharmacia Biotech Brasil Ltda, São Paulo, Brazil) to each well, and the culture was continued for an additional 2 hours. Subsequently, the thymidine was removed, the cells were washed twice with phosphate-buffered saline, and DNA was precipitated with 5% trichloroacetic acid. 300 µl of 0.2 N NaOH/0.3% n-lauroylsarcosine Na salt solution (Sigma) were added at each well, and aliquots of 60 µl were placed at 3 ml of liquid scintillation. Samples were protected from light for 2 hours and then analyzed by a Beckman beta scintillation counter model LS6200 (Beckman Instruments, Palo Alto, CA, USA). Results were expressed as counts per minute (CPM).

### 2. Flow cytometry

We analyzed the extracellular expression of Alkaline Phosphatase and the intracellular staining of Osteocalcin and MCP-1. Briefly, after dissociation, bone cells (0.5×10^6^) were placed in cytometry tubes, washed, centrifuged and incubated with optimal amounts of monoclonal antibodies to detect anti-human bone, liver and kidney alkaline phosphatase −10 µg/ml (US Biological, MA, USA), Phycoerythrin (PE) -conjugated anti-human osteocalcin −0.25 µg/ml (R&D Systems Inc, MN, USA), and anti-human CCL2/MCP1 −2,5 µg/ml (R&D Systems Inc, MN, USA), for 30–45 minutes at 4°C in darkness. For the intracellular staining, cells were first fixed with paraformaldehyde fixative (4% paraformaldehyde in 10 mM phosphate buffered saline) and permeabilized with SAP buffer (0.1% saponin, 0.05% NaN_3_ in Hanks Balanced Salt solution HBSS). When necessary, cells were incubated for 30 minutes at 4°C in darkness with a secondary FITC-conjugated antibody to allow binding to the primary antibody. As negative control, we used for each patient an isotype control in the same conditions as for extracellular or intracellular staining. 10^4^ events were normally acquired and bone cells were distinguished using light scatter characteristics [forward-scattered light (FSC) that is proportional to cell-surface area or size and side-scattered light (SSC) that is proportional to cell granularity or internal complexity]. Data were analyzedusing a FACSCalibur flow cytometer (Becton Dickinson, San Diego, USA) and a CellQuest Software (Becton Dickinson, San Diego, USA), and the results were expressed as the percentage of positive cells and as mean fluorescence intensities (MFIs), corrected for background fluorescence.

### Histomorphometry and immunohistochemistry analysis

Bone material designated to histomorphometry and immunohistochemistry was fixed in 70% ethanol and processed according to Malluche and Faugere [59]. Using a Polycut S equipped with a tungsten carbide knife (Leica, Heidelberg, Germany), undecalcified bone were cut into sections of 5 µm and 10 µm in thickness. Static histomorphometric data were obtained using a semiautomatic image analyser and the software Osteomeasure (Osteometrics Inc., Atlanta, GA, USA). Unstained 10 µm slices were obtained for analysis of dynamic parameters under a microscope with ultraviolet light.

The parameters analyzed were in accordance with the standards set by the American Society for Bone and Mineral Research [60]. The histomorphometric static parameters analyzed were: bone structure, as defined by measuring trabecular volume (BV/TV) (%), trabecular thickness (Tb.Th) (µm), trabecular separation (Tb.Sp) (mm) and trabecular number (Tb.N) (/mm); bone formation, as defined by osteoid volume (OV/BV) (%), osteoblast surface (Ob.S/BS) (%), osteoid thickness (O.Th) (µm) and osteoid surface (OS/BS) (%); and bone resorption, as defined by eroded surface (ES/BS) (%) and osteoclast surface (Oc.S/BS) (%). Kinetic parameters were: mineralizing surface (MS/BS) (%), mineral apposition rate (MAR) (µm/day), bone formation rate (BFR/BS) (µm^3^/µm^2^/day) and mineralization lag time (MLT) (days). The biopsy samples presented a tissue area of 9.2–54.0 mm^2^. Three samples presented a tissue area <30 mm^2^.

We performed an immunohistochemistry method for detection of proteins on bone sections embedded in methyl metacrylate [61]. Briefly, bone sections were deacrylated in a mixture of xylene and chloroform, rehydrated in graded alcohol solutions, submitted to a quick decalcification with 1% acetic acid, and treated with 0.1% Tween 20. Endogenous peroxidase activity was inhibited by a mixture of 3% hydrogen peroxide in methanol. Protein Block (DAKO Corporation, California, USA) was used to block nonspecific bindings. Sections were incubated overnight at 4°C in a humidified chamber using the primary goat monoclonal antihuman antibodies Osteoprotegerin (OPG) (1∶80) and Receptor Activator of NF-κB ligand (RANKL) (1∶150) (Santa Cruz Biotechnology, Santa Cruz, CA, USA). After incubation with the primary antibody, sections were incubated with the biotinylated secondary antibodies (*Biotinylated Link Universal,* DAKO Corporation, California, USA) and with the *Streptavidin-HRP* (DAKO Corporation, California, USA). Antigen-antibody complexes were visualized using a 3-amino-9-ethylcarbazole as chromogen substrate (AEC). The sections were counterstained with Mayer’s hemalum solution (Merck, Darmstadt, Germany). Simultaneous negative controls were carried out by omitting primary antibody in all sections. As positive controls, we used bone sections from patients with Chronic Kidney Disease-Mineral and Bone Disease (CKD-MBD). Each bone sample was entirely evaluated in a light microscopy at a magnification of 200×. Immunopositivity was determined as the percentage of positive osteocytes compared with the total osteocyte number in each biopsy.

Controls for histomorphometric static parameters and immunohistochemistry were 15 female age-matched healthy individuals from a large Brazilian bone database [62]. Most subjects had been victims of gunshot or knife wounds, trauma, or traffic accidents and were not known to have any disease. Dynamic parameters were compared to 29 healthy female subjects from Melsen et al (mean age 29 years) [63].

### Statistical Analysis

Results are expressed as mean ± stand deviation, median (25–75 IQR) or n/%. For comparisons between groups, we used chi-square and Mann–Whitney tests, as appropriate. Correlations between variables were determined using the Spearman correlation coefficient. Urinary MCP-1 was log transformed and used as a dependent variable in linear regression models. Analyses were performed using the SPSS v 13.0 software. Tests were all two-sided and *p* value <0.05 was considered as significant.

## Results

The results of the clinical, biochemical and histological classification of LN are described in the table 1. LN patients presented a mean age of 29.5±10 years. The mean proteinuria was 4.7±2.9 g/day and the estimated GFR was 37(31–87) ml/min/1,73 m^2^. Proliferative Lupus Nephritis was observed in 86.6% of the patients. The mean cumulative prednisone dose was 1953±765 mg in 34±12 days, reflecting that our patients presented a high activity and received an aggressive treatment. All patients presented vitamin D insufficiency (25-hydroxyvitamin D_3_ = 9.9±4.4 ng/ml, range 4–20).

**Table 1:** Clinical, biochemical and histological characteristics of lupus nephritis patients.

		mean±sd, median(25–75 IQR) or n/%	Range (when appropriate)
**Age (years)**		29.5±10	18–47
**SLEDAI**		21±6	14–32
**Cumulative glucocorticoid dose (mg of prednisone)**		1953±765	950–3720
**Time of glucocorticoid use (days)**		34±12	16–62
**Use of angiotensin-converting enzyme (ACE) inhibitors or angiotensin receptor blockers**		8/53.3%	–
**Hemoglobin (g/dL)**		9.6±1.1	7.4–11.9
**Class of Lupus Nephritis**	**II**	1/6.7	–
	**III**	3/20	
	**IV**	10/66.6	
	**V**	1/6.7	
**Positive Anti-DNA**		11/73.3	–
**Decreased C4 complement**		13/86.7	–
**24 hour proteinuria (g/day)**		4.7±2.9	0.8–9.7
**Serum albumin (g/dL)**		2.4±0.8	1.0–3.9
**Total cholesterol (mg/dL)**		306±130	152–679
**Triglycerides (mg/dL)**		268±111	85–480
**Serum creatinine (mg/dl)**		1.67 (0.89–1.83)	0.57–4.85
**Estimated GFR (ml/min/1.73** **m^2^)**		37 (31–87)	12–155
**Total calcium (mg/dL)**		8.2±0.8	6.6–9.5
**Phosphorus (mg/dL)**		4.9±1.2	3.2–7.6
**Alkaline phosphatase (U/L)**		58 (45–85)	42–259
**25(OH)D (ng/ml)**		9.9±4.4	4–20
**PTH (pg/ml)**		73 (29–102)	14–196

Legend: SLEDAI (Systemic Lupus Erythematosus Disease Activity Index); GFR (Glomerular Filtration Rate estimated by the MDRD simplified formula); 25(OH)D (25-hydroxyvitamin D_3_); PTH (parathyroid hormone).

Table 2 describes the clinical and biochemical characteristics of the patients and controls. There were no differences in age and body mass index (BMI). LN patients presented lower levels of estimated GFR and 25(OH)D. They also presented significantly higher levels of IL-6, TNFα and MCP-1, reinforcing that they were on high disease activity. Patients also presented higher levels of serum deoxypyridinoline.

**Table 2:** Clinical and biochemical features of lupus nephritis patients and controls.

	Lupus Nephritis (n = 15)	Controls (n = 15)	p
**Age (years)**	29.5±10	31.7±6.4	0.25
**Body Mass Index (BMI) (Kg/m^2^)**	24±3	24±4	0.74
**Estimated GFR (ml/min/1.73** **m^2^)**	37 (31–87)	90 (73–100)	0.009
**25(OH)D (ng/ml)**	9.9±4.4	24.3±6.2	<0.001
**Serum MCP1 (pg/ml)**	18.3 (16.3–39.9)	11.1 (10.3–13.4)	<0.001
**Urinary MCP1 (pg/mg Cr)**	1594 (595–2447)	177 (113–267)	<0.001
**Serum IL-6 (pg/ml)**	5.8 (1.9–11.3)	1.2 (1.1–1.4)	0.002
**Serum TNFα (pg/ml)**	9.1 (8.2–19.9)	5.6 (5.2–6.7)	<0.001
**Serum deoxypyridinoline (nmol/L)**	9.1 (5.4–15.5)	3.8 (3.5–4.9)	<0.001

Legend: Results are expressed as mean ± standard deviation, median (25–75 IQR) or n/%. GFR (Glomerular Filtration Rate estimated by the MDRD simplified formula); 25(OH)D (25-hydroxyvitamin D_3_); MCP1 (Monocyte Chemoattractant Protein-1); IL-6 (Interleukin 6); TNFα (Tumor Necrosis Factor α).

**Figure 1:**
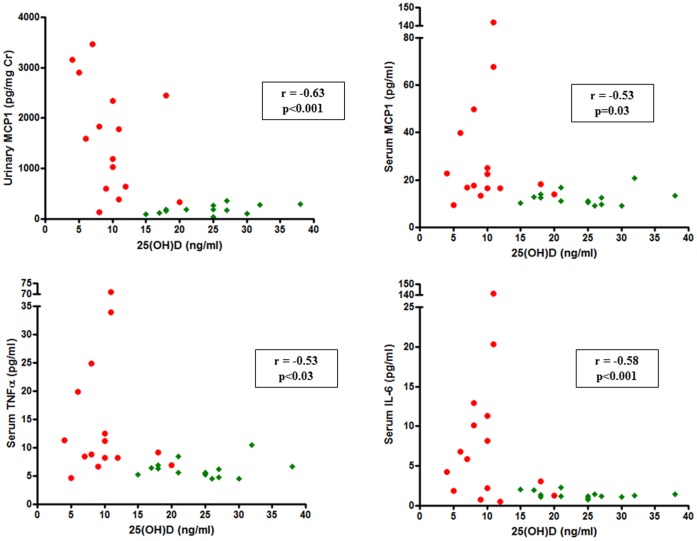
Correlation between 25(OH)D levels and inflammatory markers. Legend: The box contains Spearman’s correlation coefficient and *p* value. Green diamonds and red circles represent controls and LN patients, respectively.

The levels of 25(OH)D correlated negatively with serum MCP-1 (r = −0.53, p = 0.003), IL-6 (r = −0.58, p<0.001), TNFα (r = −0.53, p = 0.003) and deoxypyridinoline (r = −0.59, p<0.001), and urinary MCP-1 (r = −0.63, p<0.001) (figure 1). We also observed a positive correlation between urinary MCP-1 and serum deoxypyridinoline (r = 0.53, p = 0.004).

Table 3 describes the results of the Linear regression analysis. The correlation between urinary MCP-1 and 25(OH)D remained unchanged even after adjusted for age, BMI and estimated GFR (beta coeff. −0.585, 95% CI −0.13 to −0.04, p = 0.001). The SLEDAI score, the period of use and the cumulative dose of glucocorticoid were not correlated with any of the studied variables.

**Table 3:** Linear regression models on Log Urinary MCP1 levels.

	Beta	Se	95% CI	p
**25(OH)D (ng/ml)**	−0.585	0.02	−0.13– −0.04	0.001
	**Beta**	**Se**	**95% CI**	**p**
**25(OH)D (ng/ml)**	−0.387	0.02	−0.11– −0.01	0.03
**Age (years)**	−0.263	0.02	−0.49–0.01	0.10
**Body Mass Index (BMI) (Kg/m^2^)**	0.133	0.06	−0.08–0.19	0.39
**Estimated GFR (ml/min/1.73** **m^2^)**	−0.360	0.01	−0.02–0.01	0.04

Legend: 25(OH)D (25-hydroxyvitamin D_3_); GFR (Glomerular Filtration Rate estimated by the MDRD simplified formula).

### Osteoblast culture

We obtained bone cells from biopsy fragments of ten patients. There were no differences between NL patients and age-matched female donor organ’s controls in osteoblast proliferation measured by incorporation of thymidine (82.22±48.43 *vs* 56.07±23.73 CPM, respectively, p = 0.21).

Table 4 shows results from flow cytometry of cultured bone cells. Osteoblasts isolated from LN patients presented a significantly higher expression of MCP-1 (32.0±9.1 *vs* 22.9±5.3 MFIs, p = 0.01), while no differences were observed in the expression of osteocalcin or alkaline phosphatase.

**Table 4:** Flow cytometry of cultured osteoblast: labeling of MCP-1, Osteocalcin and Alkaline Phosphatase of Lupus Nephritis patients and controls.

	Lupus Nephritis (n = 10)	Controls (n = 9)	p
**MCP-1 (%)**	95.1±2.3	92.9±4.3	0.24
**MCP-1 (MFIs)**	32.0±9.1	22.9±5.3	0.01
**Osteocalcin (%)**	12.25±7.13	4.66±3.69	0.007
**Osteocalcin (MFIs)**	1.77±0.61	1.63±0.50	0.63
**Alkaline Phosphatase (%)**	49.8±17.2	40.5±15.1	0.22
**Alkaline Phosphatase (MFIs)**	20.9±12.7	16.3±10.9	0.33

Legend: Results are expressed as mean ± standard deviation. MFIs (mean fluorescence intensities); % (percentage of positive cells).

We observed no differences between NL patients and controls concerning the percentage of positive MCP-1 and Alkaline Phosphatase cells. There was an increased percentage of osteocalcin positive cells in LN patients (12.25±7.13 *vs* 4.66±3.69%, p = 0.007) (Table 4).

### Bone histomorphometry

Results of the bone histomorphometry analysis of the patients and controls are summarized in table 5. We did not observe any difference between both groups concerning to structural parameters. LN patients presented a significantly reduced osteoid volume (OV/BV), thickness (O.Th) and surface (OS/BS). The mineralization surface (MS/BS) and bone formation rate (BFR/BS) were also reduced in LN patients. The patients also presented higher eroded surface (ES/BS) and osteoclast surface (Oc.S/BS).

**Table 5:** Histomorphometric parameters of LN patients and controls.

	Lupus Nephritis (n = 15)	Controls (n = 15) [^62^] (n = 29) [^63^]	p
***STRUCTURAL***			
BV/TV (%)	26.0±6.8	26.4±5.0	0.98
Tb.Th (µm)	130.7±23.7	122.8±21.2	0.49
Tb.Sp (µm)	390.4±110.9	346.8±63.2	0.42
Tb.N (/mm)	1.99±0.39	2.15±0.32	0.22
***FORMATION***			
OV/BV (%)	0.19 (0.08–0.38)	1.63 (1.10–2.96)	**<0.001**
Ob.S/BS (%)	0.68 (0.20–2.65)	0.80 (0.20–2.66)	0.69
O.Th (µm)	2.85±1.04	11.4±2.1	**<0.001**
OS/BS (%)	3.6 (2.1–8.9)	8.9 (7.2–16.5)	**0.03**
***MINERALIZATION***			
MS/BS (%)	2.2±1.6	12.0±5.0	**<0.001**
MAR (µm/day)	0.44±0.23	0.64±0.10	0.41
BFR/BS (µm^3^/µm^2^/day)	0.01±0.01	0.07±0.03	**<0.001**
MLT (days)	28.8±12.8	23.7±2.7	0.71
***RESORPTION***			
ES/BS (%)	9.78 (5.02–11.80)	2.00 (1.30–3.30)	**<0.001**
Oc.S/BS (%)	0.54 (0.19–0.69)	0.02 (0–0.10)	**<0.001**

Legend: Results are expressed as mean ± standard deviation, median (25–75 IQR). Trabecular volume (BV/TV) (%); trabecular thickness (Tb.Th) (µm); trabecular separation (Tb.Sp) (µm); trabecular number (Tb.N) (/mm); osteoid volume (OV/BV) (%); osteoblast surface (Ob.S/BS) (%); osteoid thickness (O.Th) (µm); osteoid surface (OS/BS) (%); mineralizing surface (MS/BS) (%); mineral apposition rate (MAR) (µm/day); bone formation rate (BFR/BS) (µm^3^/µm^2^/day); mineralization lag time (MLT) (days); eroded surface (ES/BS) (%); osteoclast surface (Oc.S/BS) (%).

### Immunohistochemistry

**Figure 2:**
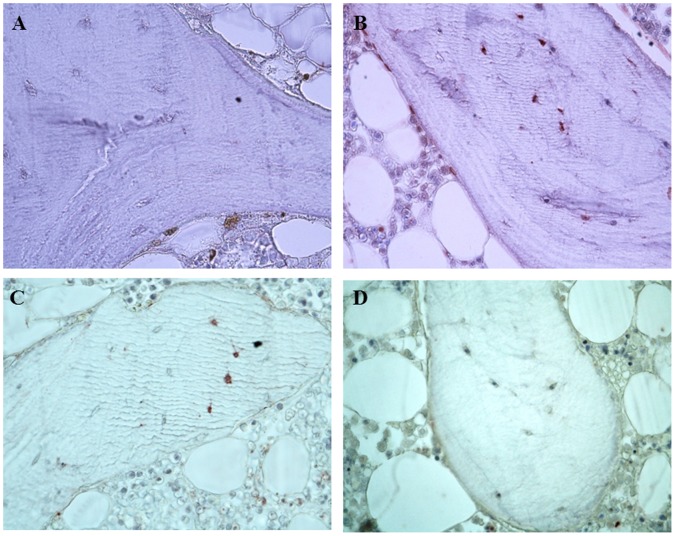
Immunohistochemistry for OPG and RANKL in bone biopsies. Legend: Positive staining appears as brown color (X200). LN patients presented a significantly lower immunostaining for OPG (A) when compared to controls (B). A stronger labeling for RANKL in osteocytes was observed in LN patients (C) when compared to healthy subjects (D).

Immunohistochemistry on patient’s bone specimens demonstrated a reduced immunostaining for OPG (0.61±0.82 *vs* 1.08±0.50%, p = 0.003), and an increased expression of RANKL (1.76±0.92 *vs* 0.41±0.28%, p<0.001) when compared to controls (figure 2).

## Discussion

Our study show that newly diagnosed lupus nephritis patients presented a reduced bone formation and mineralization, associated with an increased bone resorption. Similar findings have already been related to glucocorticoid-induced bone disease. As previously mentioned, it is not clear the minimum dose and duration of glucocorticoid therapy that could significantly impact bone health. Most histomorphometric studies evaluated patients submitted to glucocorticoids for 6 months to 35 years, with current doses of 5 to 12.5 mg of prednisone [64]–[66]. Despite a limited time of exposure, our patients received high doses of corticosteroids. Thus, it is not possible to exclude the influence of glucocorticoids on our findings.

Vitamin D insufficiency was observed in all of our patients and might also play a role in the bone metabolism disturbance. Reduced calcitriol levels are thought to impair bone mineralization, by limiting the amount of available calcium and phosphorus, and favor bone resorption, by stimulating PTH synthesis and secretion [67], [68]. Moreover, recent studies suggest that 25-hydroxivitamin D levels might also influence osteoblast proliferation and functions (through autocrine pathways) [69]–[72].

Decreased vitamin D levels in SLE patients have been related to several factors. Ruiz-Irastorza et al shown that photosensitivity [odds ratio (OR) 3.5] and photoprotection (OR 5.7) predicted vitamin D insufficiency and deficiency, respectively [73]. It is noteworthy that our patients were all newly diagnosed, and although some required a short hospitalized time, their blood samples were collected at hospital admission and none of them have been previously guided to avoid sun exposure.

Renal involvement is also related to a higher risk for vitamin D deficiency in SLE patients [42], [43]. Chronic kidney disease (CKD) may lead to reduced 1.25-dihydroxyvitamin D3 levels by limiting the amount of 1α hydroxylase or suppressing its action through increased fibroblast growth factor-23 [74]. In our patients, reduced GFR was clearly the result of glomerular acute inflammation and not a CKD condition. On follow-up, six months after diagnosed, LN patients presented an estimated GFR of 81(49–121) ml/min/1,73 m^2^ and only one of them was still in haemodialysis, reinforcing that at baseline our patients presented primarily an acute kidney injury related to SLE activity.

Proteinuria may also lead to reduced levels of 25(OH)D due to urinary losses of vitamin D binding proteins [75], [76], with recognized implications on bone metabolism [77], [78]. Using a study protocol similar to ours, Dias et al from our laboratory previously demonstrated that primary Glomerulopathies (PG) patients [69] presented a reduced bone formation and an enhanced bone resorption. When we compared our LN patients to those PG female patients (with author’s permission), we observed that, despite a similar age, BMI, estimated GFR and proteinuria (data not shown), LN patients presented significantly lower 25(OH)D levels (9.9±4.4 *vs* 18.4±11.1 ng/ml, p = 0.04), probably contributing to an increase in PTH levels [73 (29–102) *vs* 19 (10–26) pg/ml, p = 0.003]. These findings support the hypothesis that several factors, besides proteinuria and renal dysfunction, may influence the vitamin D status in SLE.

Finally, despite moderate correlation coefficients, 25(OH)D levels were statistically correlated to all inflammatory mediators studied (IL-6, TNFα, and MCP1). The negative correlation between vitamin D and disease activity has already been demonstrated not only in SLE patients, using SLEDAI and Physicians’ Global Assessment indexs [28], [79], [80], but also in rheumatoid arthritis [81], supporting the potential immune modulatory effects of vitamin D.

Cells obtained from the trabecular bone surfaces of LN patients and controls largely expressed alkaline phosphatase, but presented a low expression of osteocalcin, pointing to characteristics of pre-osteoblast phenotype. Patient’s osteoblasts presented a significantly higher expression of MCP-1. Recent studies reiterate a cross-talk between bone cell lineages and resident tissue macrophages, as well as Treg cells [82]. Since urine levels and renal tissue expression of MCP-1 has been extensively related to lupus nephritis activity, we can speculate that resident MCP1 primed bone macrophages could stimulate a MCP1 osteoblast phenotype. This speculation needs experimental confirmation.

Interestingly, it has already been shown that this chemokine is produced by osteoblasts and, in response to RANKL, might be expressed by osteoclast precursors, acting as a recruiter of monocytes and other osteoclast precursors [83]–[86]. MCP-1 expression has already been detected at sites of tooth eruption, bone metastasis and Rheumatoid Arthritis, being regulated by inflammatory cytokines such as TNF-α and Interleukin (IL)-1b and hormones, such as PTH [84], [85], [87]–[90]. Therefore, augmented MCP-1 related to LN activity may help to explain the increase in osteoclast activity and bone resorption detected in our patients.

LN patients presented a reduced immunostaining for OPG and an increased expression of RANKL, suggesting that OPG/RANKL system may be involved in the increased bone resorption observed in these patients. As previously mentioned, the OPG/RANKL system, composed by TNF superfamily members, is a final signaling pathway involved in bone resorption modulation. RANKL is a transmembrane protein produced by osteoblastic lineage cells and activated T cells. Besides immunomodulatory effects, binding of RANKL to its specific receptor RANK stimulates osteoclast formation, fusion, differentiation, activation and survival, leading to enhanced bone resorption and loss. Noteworthy, RANKL expression by the osteoblasts might be up-regulated by several cytokines, as previously mentioned [35], [91]–[93].

On the other hand, OPG acts as a solube inhibitor that prevents RANKL binding with RANK, preventing osteoclast differentiation and activation, and promoting osteoclast apoptosis. The production of OPG by a variety of tissues (such as cardiovascular system, bone and immune cells) is also modulated by various cytokines, hormones and drugs. To notice, glucocorticoids, PTH and basic fibroblast growth factor might suppress OPG expression [92], [93].

Our study presents several limitations. First, we studied acute and severely ill patients. In this scenario it is very difficult to isolate the possible influence of each factor involved in the pathogenesis of bone disease. Second, the limited time of disease might have prevented us to observe a significant modification on structural parameters of the bone biopsies. Third, osteoblast proliferation was studied in vitro. This method, although standardized and broadly reproducible, does not reflect local and systemic factors that may influence cell proliferation in vivo.

In conclusion, newly diagnosed lupus nephritis patients present a significant disturbance in bone remodeling, characterized by an impaired bone formation and mineralization, associated with an increase in bone resorption. We believe that glucocorticoid use and vitamin D insufficiency, observed in all of our patients, might have influenced the bone disturbance. Bone tissue showed a reduced immunostaining for OPG and an increased expression of RANKL, suggesting that OPG/RANKL system may be involved in the increased bone resorption. The positive correlation between urinary MCP-1 and serum deoxypyridinoline levels, as well as the increased expression of MCP-1 by cultured bone explants cells suggests that inflammatory background may contribute to bone resorption in LN patients.
